# The role of evaluatively conditioned stimuli in iconic memory

**DOI:** 10.1007/s00426-024-02024-w

**Published:** 2024-09-25

**Authors:** Alexandra E. Clausen, Florian Kattner

**Affiliations:** 1 Institute of Psychology, Technical University of Darmstadt, Alexanderstr. 10, 64283 Darmstadt, Germany; 2 Institute for Mind, Brain and Behavior, Health and Medical University, Potsdam, Germany

**Keywords:** Evaluative conditioning, Valence, Iconic memory, Visual processing

## Abstract

In the present study, an attempt was made to replicate results found about the influence of valence on prioritisation and decay in iconic memory. Hereby, the evaluative conditioning effect was used to induce valence for formerly neutral stimuli. The effect is gained by pairing neutral stimuli with either positive, negative, or neutral images in a conditioning phase. Afterwards, the conditioned stimuli acted as targets in an iconic memory test. In the iconic memory test, targets were presented together with seven other stimuli on a circular placement on the screen for a short time. A cue delayed by either 17, 68, 221, 493, or 1003 ms pointed at the target to be reported. Participants rated the targets before and after the conditioning phase. In addition, the affective and neutral images used in the pairing procedure were rated at the end of the experiment. While no significant change in rating could be observed for the conditioned targets, a significant effect of conditioned valence was still present in the response times and the accuracy of the iconic memory test. Participants reacted the quickest in response to a cue for positively conditioned targets compared to neutral or negatively conditioned targets. Accuracy was highest for positively conditioned targets and was lowest for negatively conditioned targets. Unlike in prior studies, slower decay of information in iconic memory for negative targets was not revealed. Further research should be conducted to identify reasons for this inconsistency.

Being in traffic, one might have to react quickly to unexpected events in the environment. The situation with children playing near the road with a ball suddenly rolling on the road is a well-liked example in a German driving school (e.g., driving licence question 1.1.02–112, retrieved from autovio.de on 22.02.2024). A quick reaction is required, which is informed by quick visual and cognitive processing of the available information. Characteristics like colour and motion (Wolfe & Horowitz, 2017) might have guided attention to the ball quicker and thereby decreased the necessary reaction time to stop safely in front of the ball. Particularly in the case of danger or threat, it is an evolutionary advantage to be able to process the advancing threat and react quickly (Öhman & Mineka, 2001). Certain animals, like spiders or snakes, usually induce a negative feeling or threat and are, therefore, seen quicker as animals not perceived to be dangerous.

Öhman, Flykt, and Esteves (2001) observed faster search times when participants were asked to look for negative targets like snakes and spiders among mushrooms or flowers. It appears that not only visual attention in visual search is affected by the affective value of snakes and spiders but also the recall and decay from iconic memory. Kuhbandner, Spitzer, and Pekrun (2011) observed slower decay of negative stimuli like guns, skulls, spiders, and scorpions in iconic memory compared to neutral or positive stimuli. In addition, accuracy was highest for negative stimuli compared to accuracy for positive stimuli. The latter was higher compared to accuracy for neutral stimuli. Research in neuroscience employing electroencephalography suggests that affective content is processed differently compared to neutral content and that this starts already at a very early stage at about 90 ms of seeing an image (Carrieté et al., 2024; Mielke et al., 2021). Carrieté et al. (2024) and Mielke et al. (2021) used affective images to investigate the visual processing of negative and neutral content. Positive images were not included in the studies. Hence, it is not known if positive content is processed differently as well compared to neutral content and if this is starting at a similar early stage. According to Vuilleumier (2005), negative valence can modulate attention by enhancing the visual processing of an affective stimulus. This enhancement for affective stimuli could then affect memory processes for these stimuli (Vuilleumier, 2005).

Quinlan (2013) pointed out that observations such as those mentioned above, particularly those in visual search, could also be due to visual characteristics of the affective stimuli used. For a better understanding of whether valence is actually contributing to the observation of visual processing, one can use evaluative conditioning as a non-invasive method to induce liking or disliking of stimuli. By pairing neutral stimuli (conditioned stimuli, CSs) with affective stimuli (unconditioned stimuli, USs) a change in rating can occur, which can be defined as the evaluative conditioning effect (De Houwer, 2007). Using this method provides the advantage of being able to use stimuli, which differ barely in their visual characteristics, just enough to be discernible, but can be changed in their perceived valence.

What is leading to the evaluative conditioning effect is still an open question (see e.g. Hütter, 2022). Some see evaluative conditioning as a product of automatic associations (e.g., Baeyens et al., 2001; Walther, 2002), while for others the creation of propositions is key to see any changes in ratings (De Houwer, 2009). More and more researchers now think that a mixture of both, automatic and propositional learning, is at play when evaluative conditioning takes place (e.g., Gawronski & Bodenhausen, 2011; Hu et al., 2017; Hütter, 2022).

Different methods of the pairing procedure of CSs and USs (e.g., side by side or one after the other; Gast et al., 2016) can contribute to the ongoing discussion, as most theories developed based on experiments (Hütter, 2022). So far, it is known that a visible presentation of the CS-US-pairings, as well as the reduction of distractions, appears to be necessary for participants to be fully aware of the pairings and be able to memorise these (Heycke & Stahl, 2020; Ingendahl et al., 2024; Kattner, 2012; Stahl et al., 2024). Missing correct memory for CS-US pairings can lead to opposite evaluations of CSs (Alves & Imhoff, 2023). Instructions should be carefully phrased and any possible context considered, as either can lead to opposite ratings than the presented US valence (Fan et al., 2021; Hütter & Sweldens, 2018). Fan and colleagues (2021) observed that made-up vaccination brands were seen as more positive after being paired with negative USs compared to when paired with neutral USs. This effect was reversed when the cognitive load was increased by adding a secondary task.

The evaluative conditioning effect can be observed in explicit ratings, but also in implicit measures like response times in the Implicit Association Test (Hofmann et al., 2010). Observing an evaluative conditioning effect in implicit measures likely indicates retrieval of associative information, while explicit measures like ratings may be more reflective of propositional reasoning (Hütter, 2022). Both processes likely contribute to affective learning to some extent (Hütter, 2022; McLaren et al., 2014), but one process is likely to be more prominent than the other. If we see differences due to conditioned valence in the iconic memory test performance, this could be explained by higher attention to CSs due to the affective learning procedure, as an increased associative strength can lead to heightened attention to that stimulus (Mackintosh, 1975). Associative learning depending on the predictability of the US would not be able to explain any differences, as according to the Rescorla-Wagner model the salience of the CSs is not changed (Yau & McNally, 2023). An effect of conditioned valence should be observed in an iconic memory test provided it boosts sensory and cognitive processes in a similar way as affective valence does as mentioned above (e.g., Carrieté et al., 2024).

While Kuhbandner, Spitzer, and Pekrun (2011) observed the highest accuracy for negative stimuli in an iconic memory test, this was the opposite in experiments conducted by Kattner and Green (2019), who used the evaluative conditioning effect to induce different valence levels. Kattner and Green (2019) investigated the effect of valence using monochrome drawings of everyday objects, which were paired with affective images in a conditioning phase before using these objects in an iconic memory task designed similar to the iconic memory task in Kuhbandner, Spitzer, and Pekrun (2011). After a short presentation of eight stimuli in circular placement around the centre of the screen, an arrow appeared after a variable delay. Participants were asked to report which stimulus had been present at the position pointed at by the arrow. The highest accuracy was found for targets, which had been paired with positive images, whilst performance for negatively paired targets was lowest in one experiment. In another experiment, Kattner and Green (2019) observed similar results, but here also a slower decay of negatively paired stimuli was present compared to the decay in iconic memory for neutral and positively paired stimuli. Kuhbandner, Spitzer, and Pekrun (2011) and Kattner and Green (2019) observed slower decay of negative stimuli in iconic memory, but this was not consistent across all experiments by Kattner and Green (2019) where in one experiment accuracy was higher for negative CSs and in one experiment for positive CSs.

In the current study, we would like to investigate if a slightly altered method in the conditioning phase and the use of fewer CSs in the iconic memory task will lead to similar results as in Kattner and Green (2019). Provided similar results are observed, this would add another piece to the cohort of experiments replicating the evaluative conditioning effect and support the importance of valence for early visual processing across visual stimuli being either inert affective or conditioned. It is valuable to show that observations can be repeatedly made and that these observations can be made even when procedures are altered (Fletcher, 2021). The present study investigated positive valence next to negative valence, which is at this point rather rare in research on early visual processing. Potentially adding further evidence for positive valence is therefore adding to the current knowledge if valence in general affects early visual processing or if this is different depending on which valence is looked at.

In contrast to the conditioning method used by Green and Kattner (2019), CSs and USs were not shown together using alpha blending but instead one right after the other. This method is very often used in studies using evaluative conditioning (e.g., Kliegl et al., 2015). Instead of only a rating at the end of the experiment, a rating as a base rating was added at the start of the experiment. Half of the participants rated the CSs a second time right after the conditioning phase, and the other half after the second iconic memory test. This was done to see if the order would make a difference in ratings or performance in the iconic memory task. Further, an iconic memory task was added before the conditioning phase with the aim to identify if any effects observed in the second iconic memory task were based on valence due to conditioning and not due to prior perceived valence. As a further manipulation check, a rating task of the USs used in the experiment was added to confirm that suitable images were used to induce the evaluative conditioning effect.

Like Kattner and Green (2019), we investigated if the decay of CSs in iconic memory is affected by the conditioned valence using a slightly altered method in the conditioning phase. Further, we are interested if we can observe similar results of prioritisation in selection from iconic memory for each level of conditioned valence.

## Method

### Participants

For this study, 40 participants (20 women, 20 men, $\:{M}_{age}$ = 22.60, $\:{SD}_{age}$ = 6.40) were recruited and randomly assigned to either task order A or task order B equally. This resulted in twenty participants in either task order A or B, with 13 men and seven women in group A and seven men and thirteen women in group B. The recruited sample size is sufficient for observing a medium effect size for evaluative conditioning (*dz* = 0.537) using visual stimuli (Hofmann et al., 2010) with a power of (1-β) = 0.95 and a significance level of α = 0.05. Participants had to be 18 years or older and have either normal or corrected-to-normal vision. Participants did not receive any compensation for their participation.

### Design

A 3 (valence) x 5 (delay) within-subjects design was implemented for the investigation of effects of valence on iconic memory due to evaluative conditioning. The variable valence contained three levels, positive and negative valence as well as a neutral control condition. Five different delays were applied to determine any prolonged availability of stimulus information as was found in prior studies (Kattner & Green, 2019; Kuhbandner, Spitzer, & Pekrun, 2011). The delay was either 17, 68, 221, 493, or 1003 ms long. Measures included accuracy and response time. To determine the evaluative conditioning effect, a 3 (valence) x 2 (time of rating) within-subjects design was used. Changes in ratings of CSs from the first to the second time of rating should be seen for the positive and negative valence level, but not for the neutral control condition.

### Apparatus and stimuli

The experiment was conducted with a monitor (Philips 190B8, 15”, 60 Hz), a keyboard and a computer mouse. MATLAB (Mathworks, Natick, MA) with the Psychophysics toolbox 3.0 extensions (Brainard, 1997; Kleiner et al., 2007; Pelli, 1997) was used to program and conduct the experiment as well as to save the data. Participants were wearing noise-cancelling headphones (JBL E55BT) and were seated about 60 cm from the screen. The distance between participants and the monitor was marked with tape on a table.

Four CSs were used in the experiment, which each consisted of three black lines on a white background that only differed in orientation. Two were oriented towards the left and two to the right. In terms of degree, the orientations were away from an upright vertical position by 22.5°, 67.5°, 112.5°, and 157.5°. Presentation size in all phases was 2° of visual angle.

Coloured and grey images from the Open Affective Standardized Image Set (OASIS, Kurdi et al., 2017) were used as USs. Twelve images per valence level were used, which totalled 36 images overall, hence containing 12 positive images, 12 negative images, and 12 neutral images. The criteria for the image selection were as follows: positive images had to have a higher average valence rating than 5.5 (Likert-scale rating) and an average arousal rating of more than 4; negative images were chosen based on being rated on valence on average at at least below 2.5 and with an average arousal rating of higher than 4; neutral images were selected from the average valence rating range of 3.5 to 4.5 and with arousal of below 3.5. The images covered a range of topics like items, animals, landscapes, and humans to appeal to a range of people’s preferences and dislikes. With the exception of direct human depiction not being in the neutral image set, all topics were available in each image set. The images were presented at 15° of horizontal visual angle during the conditioning phase and in the US rating phase.

Practice images for the iconic memory test practice were out of a pool of 12 black animal figures on a white background. The presentation size was the same as the CSs, 2° of visual angle.

### Procedure

The guidelines of the 1964 Helsinki Declaration with all its later amendments and the APA Ethical Principles of Psychologists were followed when planning and conducting the experiment. The ethics committee of the Technical University of Darmstadt approved the design of the experiment (EK 04/2017). The tasks’ design followed those of Experiment 2a and b reported in Kattner and Green (2019) with small adjustments.

The experiment lasted about an hour and contained seven phases. The order of these phases differed at one point, which will be differentiated between by (order) A and B. All participants started the same way with the *pre-rating phase* of the CSs. This was followed by the *iconic memory practice phase* and the *iconic memory pre-test phase* before the *evaluative conditioning phase*. Then, the *evaluative conditioning phase* followed. Participants of group A rated the CSs a second time in the *post-rating phase* before completing the *iconic memory post-test phase*. Participants of group B did the *iconic memory post-test phase* right after the *evaluative conditioning phase* and then rated the CSs a second time (*post-rating phase*). All participants ended the experiment with a rating of the USs (*US rating phase*).

In the CS *pre-rating phase*, the four CSs were shown in random order, slightly above the middle of the screen. A grey line with 21 small grey bars appeared underneath the CS. On the left side, it said “like not at all” in German (“gefällt mir gar nicht”), and on the right, it said “like very much” in German (“gefällt mir sehr”). The bars were encoded as numbers, but no numbers were visible to the participants to avoid any orientation towards certain numbers. Participants used the mouse to click anywhere on the grey line, and they were encouraged to rate without too much thought based on feeling. After each rating, a blank screen was shown for 500 ms, and then a new trial started.

The *iconic memory practice* phase included 10 trials in which participants saw eight practice images on the screen for a short time and after a variable delay had to indicate which image was shown at a certain position. A fixation point was presented on its own for 750 ms in the middle of the screen. The fixation point stayed throughout one trial. On an invisible circle with a radius of 6° (Kuhbandner, Spitzer, & Pekrun, 2011) four randomly chosen practice images were placed twice, leading to eight stimuli overall on the screen. The possible positions were at 45°, 90°, 135°, 180°, 225°, 270°, 315°, and 360°, and positioning was random. The presentation lasted for 136 ms. After a variable cue delay of either 17, 68, 221, 493, or 1003 ms, an arrow appeared on the screen, pointing to one position of the possible eight. The task was to report which image had been shown at that position. Participants answered by clicking on one of the 12 practice images, which were shown in the bottom fifth of the screen in one row underneath a black line separating the answer field from the rest of the screen. A blue frame around the chosen answer indicated the successful choice and immediate feedback was presented in the centre of the screen. The feedback was “correct” in German (“Richtig!”) in green writing or “wrong” in German (“Falsch!”) in red writing and either was shown for 1000 ms. The next trial started automatically right after.

**Fig. 1:**
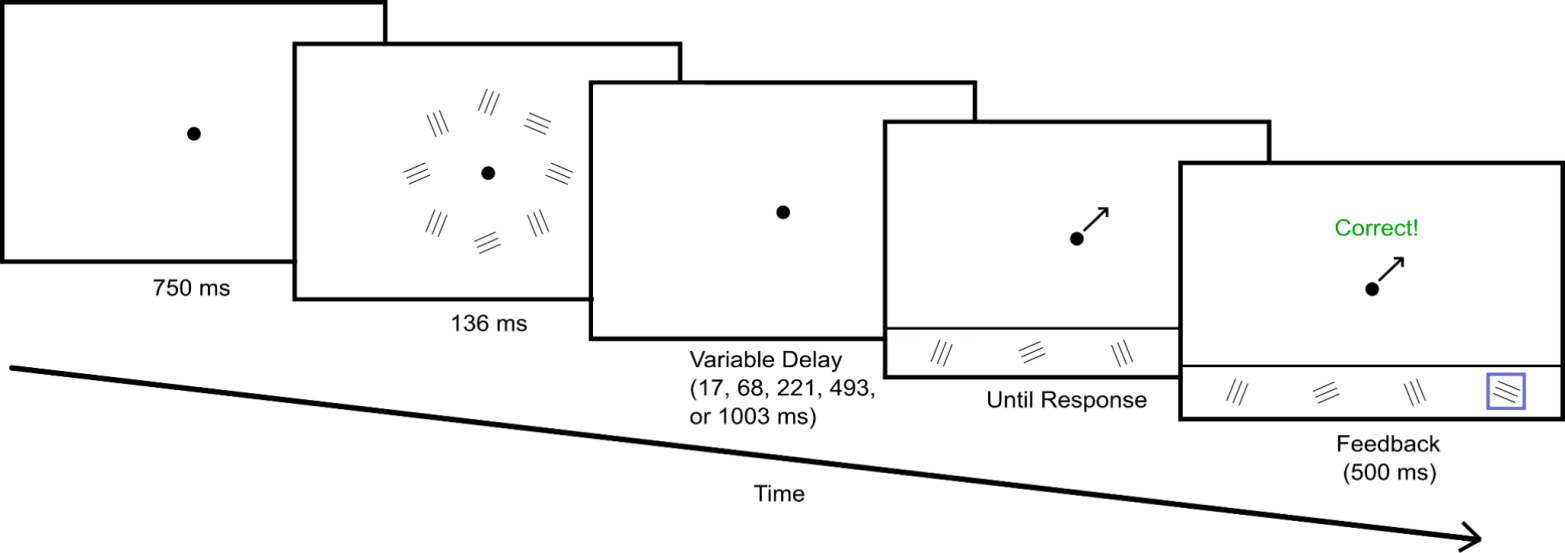
Schematic illustration of a trial of the iconic memory task *Note*. Example of a trial with the CSs as targets

The four CSs were shown twice in the *iconic memory pre-test phase*, and their positioning was random on the eight available positions in each of the five cue-delay conditions as described in the practice phase. An example of a trial can be seen in Fig. 1. The combination of the five cue delays and the four CS was repeated eight times, which led to 160 trials in this task. The order was random, and the procedure was the same as in the practice phase, but feedback was now shown for 500 ms. There was one break after 80 trials, which participants could end on their terms by clicking the left mouse button when they felt ready to continue.

**Fig. 2:**
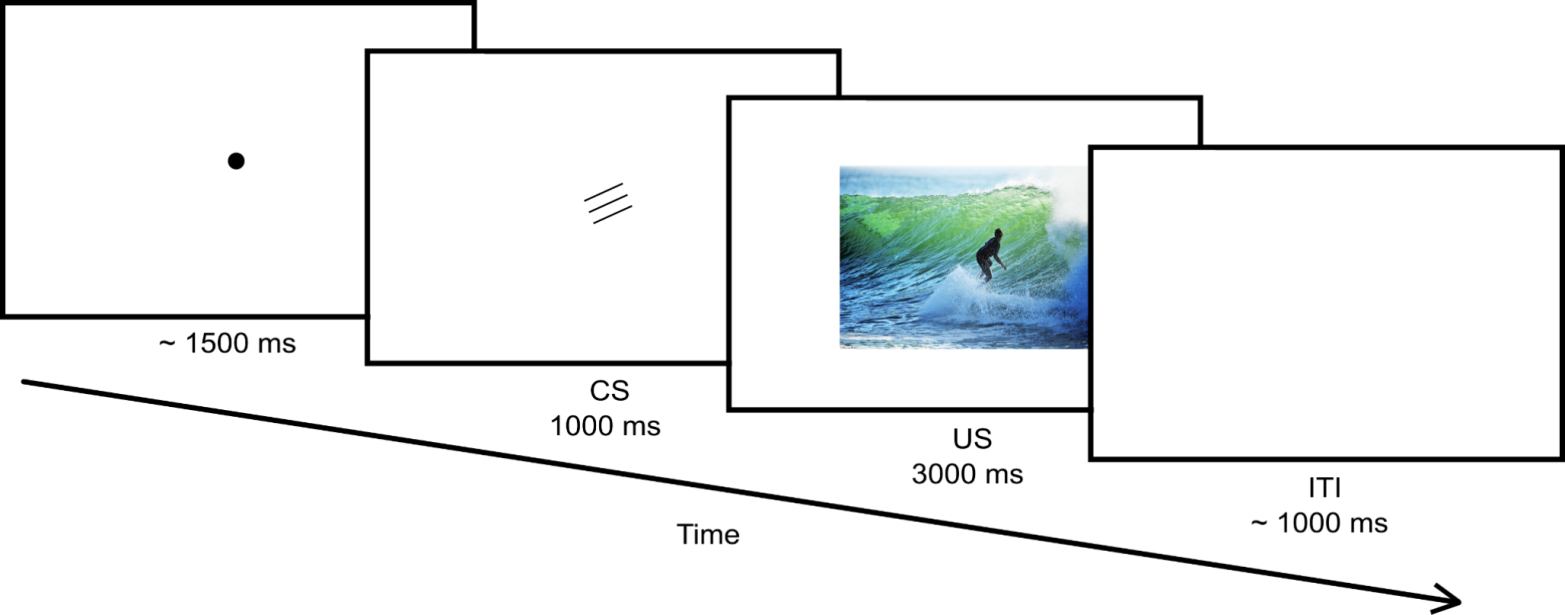
Schematic illustration of a trial of the evaluative conditioning phase *Note*. The image representing the US in this figure was not used in the experiment

The CSs were paired with the USs of their assigned valence in the *evaluative conditioning phase*. This assignment was random, with one CS being assigned to negative valence, one CS to positive valence, and the other two CSs to the neutral condition. The conditioning phase started with a fixation point for a variable period based on a normal distribution (*M* = 1500 ms, *SD* = 100 ms), which then disappeared. A CS was shown in the centre of the screen for 1000 ms, which was replaced by a US of the assigned valence set for 3000 ms. A variable inter-trial interval based on a normal distribution (*M* = 1000 ms, *SD* = 500 ms) followed, showing a blank screen. The next trial followed automatically. An example of a trial can be seen in Fig. 2. Given four CSs being paired with 12 images, there were overall 48 trials in this phase.

The *iconic memory post-test phase* followed the *evaluative conditioning phase* for group B, while group A did this after the CS *post-rating phase*. The *iconic memory post-test phase* was identical to the *iconic memory pre-test phase*. Again, all trials were in random order.

The CS *post-rating phase* was the same as the *pre-rating phase.* All CSs were shown in random order. The *US rating phase* followed the same procedure as the CS ratings. After the *US rating phase*, participants were instructed to seek the experimenter for the debriefing.

### Data analysis

First, it was tested whether participants liked and disliked images used as USs as expected by conducting an analysis of variance on US ratings for each valence level. Second, an analysis of variance on CS ratings was conducted to see if an evaluative conditioning effect could be observed. A significant interaction of the within-subjects factors valence and time of rating would indicate successful conditioning. Third, response time and accuracy were looked at for the iconic memory post-test using analyses of variance. The within-factors valence and delay were of interest to see if valence had an effect on prioritisation in recall and decay in iconic memory. Trials with a response time shorter than 50 ms were excluded from data analysis. The level of significance was set at 0.05.

## Results

All results reported are across groups, as there were no significant group differences observable due to the different order of the iconic memory post-test ($\:{M}_{Group\:A}$ = 0.40; $\:{SD}_{Group\:A}$ = 0.09;$\:\:{M}_{Group\:B}$ = 0.40; $\:{SD}_{Group\:B}$ = 0.15) and the CS post-rating ($\:{M}_{Group\:A}$ = 11.33; $\:{SD}_{Group\:A}$ = 1.66;$\:\:{M}_{Group\:B}$ = 10.64; $\:{SD}_{Group\:B}$ = 2.51). Given a significant result of the Mauchly test for sphericity (Mauchly, 1940) indicating a violation of sphericity, degrees of freedom were corrected using the Greenhouse-Geisser correction (Greenhouse & Geisser, 1959). The Benjamini-Hochberg correction was used when conducting post-hoc analysis with pairwise t-tests to correct for multiple comparisons (Benjamini & Hochberg, 1995).

### US and CS ratings

Participants perceived the chosen USs as intended for each valence level ($\:{M}_{positive}$ = 16.66; $\:{SD}_{positive}$ = 2.43;$\:\:{M}_{neutral}$ = 11.29; $\:{SD}_{neutral}$ = 1.74; $\:{M}_{negative}$ = 4.64; $\:{SD}_{negative}$ = 2.17). This was confirmed by a one-way-ANOVA on US ratings, *F*(1.45,56.58) = 263.07; *p* <.001; η_G_^2^ = 0.845. Degrees of freedom were corrected with ε = 0.725. Corrected pairwise t-tests showed that ratings for neutral images were significantly lower than for positive images (*p* <.001, *dz* = -1.620). Negative images were rated far lower than positive images (*p* <.001, *dz* = -2.930) and also lower than neutral images (*p* <.001, *dz* = -2.930).

**Fig. 3:**
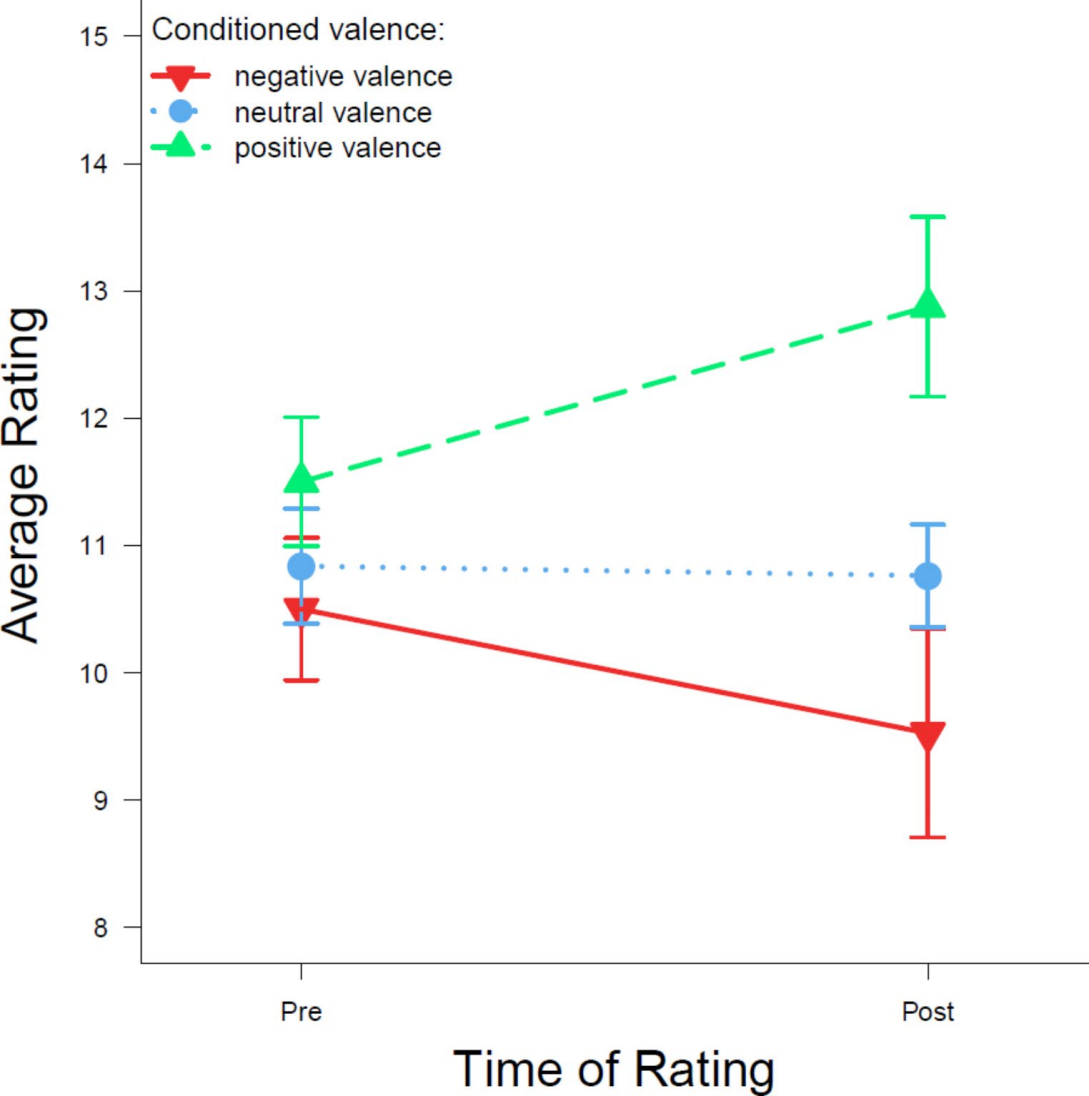
Average evaluative ratings of the different CSs in the pre- and post-rating phases *Note*. Average CS ratings for each valence level before (pre) and after (post) the evaluative conditioning phase across both groups. The rating scale ranged from 1 to 21. Error bars show the standard errors of the mean

The average pre- and post-ratings of CSs can be seen in Fig. 3. While all CSs were rated in the neutral area on average at first, it appears that some participants already rated the CS assigned to positive valence slightly more positively. When CSs were rated a second time, the average ratings tended to go in the intended direction. The first impression is supported by the results of a 3 (valence) x 2 (time of rating) analysis of variance. A significant effect of valence is present, *F*(1.71,66.63) = 5.69; *p* = .008; η_G_^2^ = 0.056, but no main effect of time of rating, *F*(1,39) = 0.05; *p* = .823. Degrees of freedom were corrected for the main effect of valence observation with ε = 0.854. There was no interaction of valence and time of rating, *F*(1.49,58.14) = 2.62; *p* = .096; degrees of freedom correction with ε = 0.745. Corrected pairwise *t*-tests revealed that overall ratings were lower for negative than for positive CSs (*p* = .005, *dz* = -0.346), and also lower for neutral than for positive CSs (*p* = .005, *dz* = -0.338). There was no significant difference between ratings for negative and neutral CSs, *p* = .187.

### Iconic memory test

On average participants correctly recognized the cued CSs in 35% of the trials in the iconic memory pre-test and in 40% of trials in the iconic memory post-test. Trials with response times faster than 50 ms were removed before analysis. This was only the case for trials in the iconic memory post-test and only for less than 0.5% of all trials. In addition, for each participant, outlier response times longer than 1.5 interquartile ranges above the third quartile of each individual’s response time distribution were removed. For the iconic memory pre-test, less than 6% of all trials accounted for outlier trials. Less than 5% of all trials in the iconic memory post-test were outlier trials.

### Valence and the post-iconic memory test

**Fig. 4:**
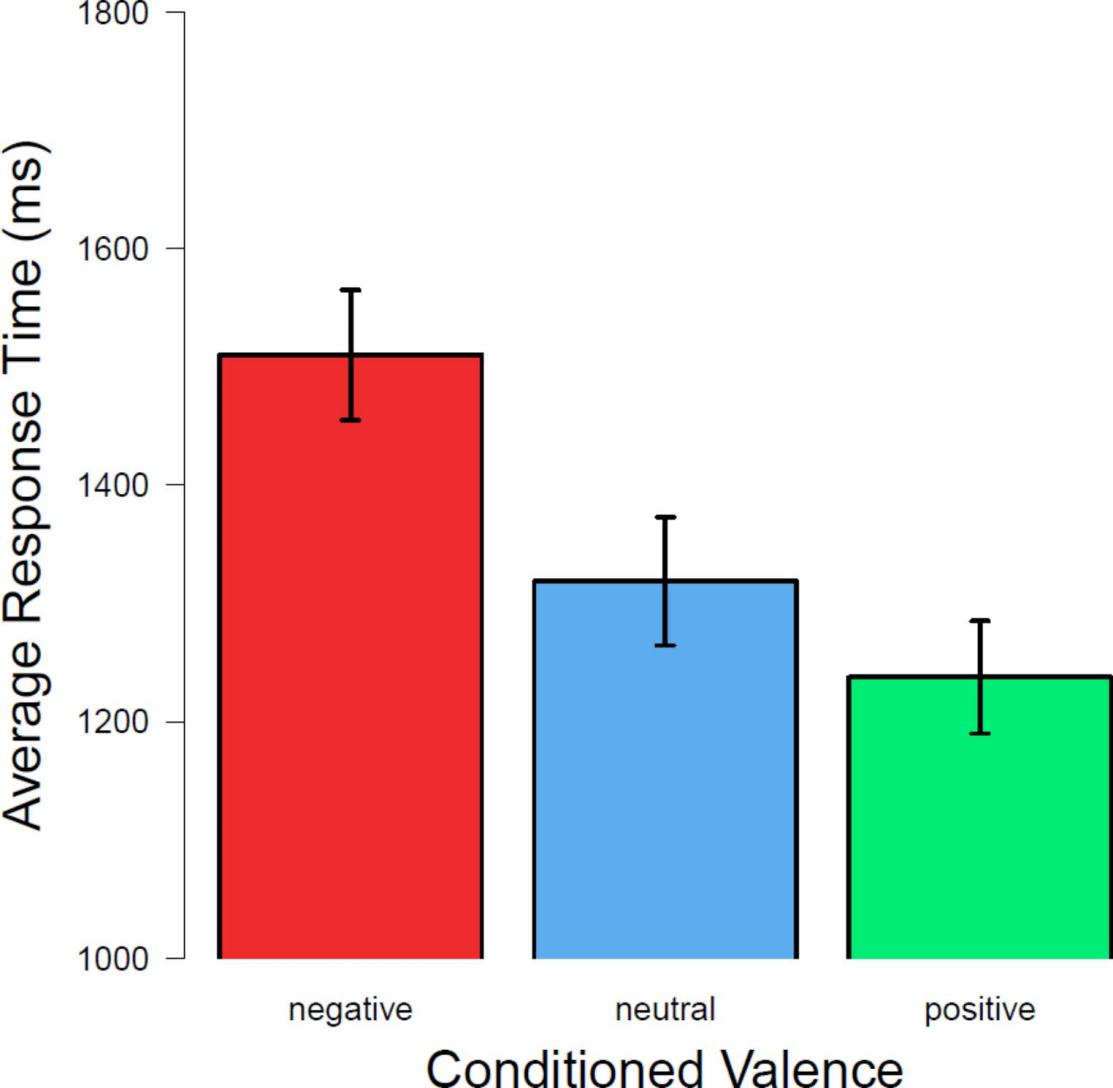
Average response times to targets of different conditioned valence in the iconic memory post-test *Note*. Average response time for each valence level in the iconic memory post-test. Only trials with correct responses are included. Error bars show the standard errors of the mean

First, data was reviewed for completeness in each dataset for each participant for each condition. Only correct trials were of interest. Twenty-one participants had data points in each condition for the analysis of response times in the iconic memory post-test. Participants were faster overall when cued for positive CSs and neutral CSs compared to negative CSs, as can be seen in Fig. 4. The average response times for each valence level in each cue-delay condition show across all delays differences between valence levels but no differences between cue-delays (see Appendix A). A 3 (valence) x 5 (delay) repeated-measures ANOVA revealed a main effect of valence, *F*(2,40) = 62.10; *p* < .001; $\:{\eta\:}_{G}^{2}$ = 0.107, but no main effect of delay, *F*(2.21,44.20) = 0.87; *p* =.437; degrees of freedom corrected with ε = 0.552. There was no significant interaction of valence and delay, *F*(4.47,89.43) =1.12; *p* =.353; correction with ε = 0.559. A post-hoc pairwise t-test revealed that response times in response to positive, neutral, and negative CSs differed significantly ($\:{p}_{positive-neutral}$ =.012, *dz* = 0.251; $\:{p}_{positive-negative}$ <.001, *dz* = 0.802; $\:{p}_{neutral-negative}$ <.001, *dz* = 0.689).

**Fig. 5:**
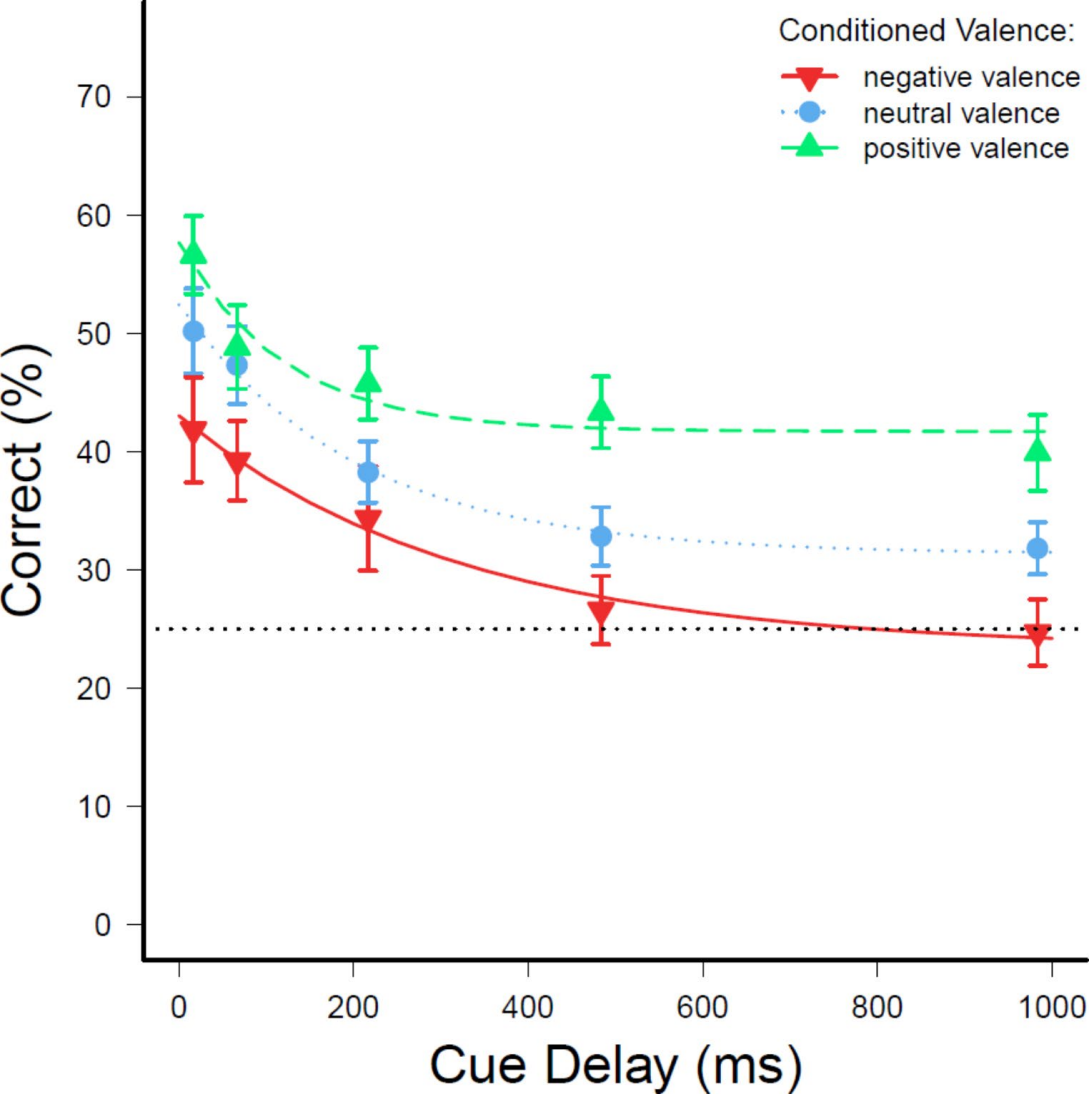
Average recognition accuracy in the iconic memory post-test as a function of the conditioned valence of the target *Note*. Average accuracy in response to CSs of each valence level in the iconic memory post-test as a function of cue-delay between target and cue arrow. Solid lines show fitted decay functions on accuracy. Error bars indicate the standard errors of the mean. The dotted line represents the chance level

Recognition accuracy in the iconic memory post-test was investigated next for the factors valence and delay. Generally, participants were more often correct in trials with a positive CS to be reported (47% correct) than for a neutral (40%) or a negative CS (33%). This pattern can also be observed throughout each delay condition, which can be seen in Fig. 5. A 3 (valence) x 5 (delay) repeated-measures analysis of variance on accuracy confirmed the first impression of a main effect of valence, *F*(2,78) = 18.83; *p* < .001; $\:{\eta\:}_{G}^{2}$ = 0.068. In addition, a main effect of delay could be observed, *F*(2.86,111.57) = 17.22; *p* <.001; $\:{\eta\:}_{G}^{2}$ = 0.092; degrees of freedom were corrected with ε = 0.715. There was no interaction of valence and delay, *F*(8,312) = 0.68; *p* =.661. Accuracy for neutral CSs was significantly lower than for positive CSs (*p* <.001, *dz* = -0.307). The amount of correct answers was also lower for negative CSs compared to for positive CSs (*p* <.001, *dz* = -0.518). Similarly, the accuracy for negative CSs was lower than for neutral CSs (*p* < .001, *dz* = -0.312). Differences in accuracy were also confirmed between the cue-delays of 17 ms and 68 ms, 17 ms and 221 ms, 17 ms and 493 ms, 17 ms and 1003 ms, 68 ms and 221 ms, 68 ms and 493 ms, 68 ms and 1003 ms, 221 ms and 493 ms, as well as 221 ms and 1003 ms (all *p* < .050). There was no difference in accuracy between the cue-delays of 493 ms and 1003 ms (*p* =.276).

**Fig. 6:**
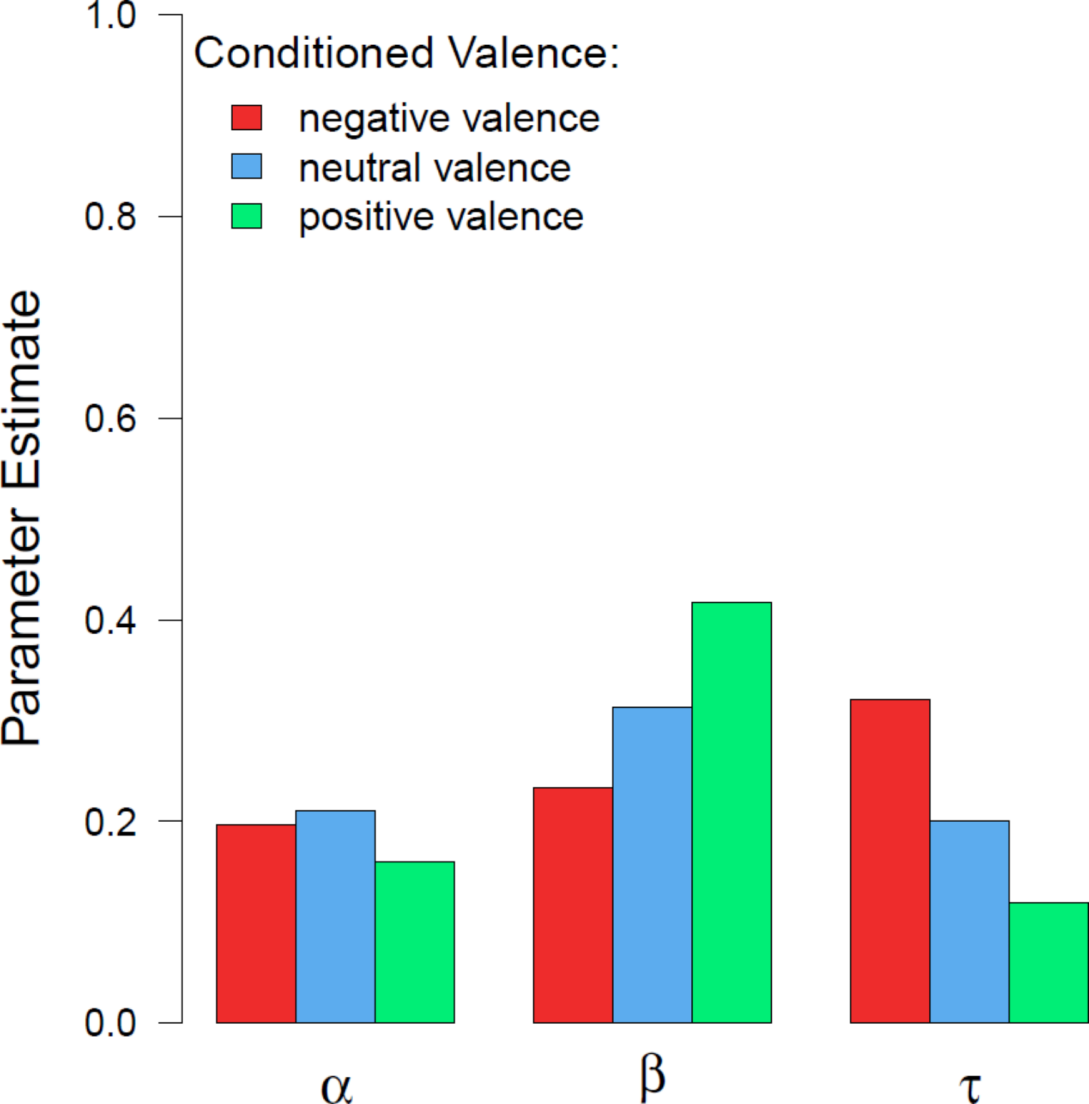
Parameter estimates *Note*. Parameter estimates of the decay function on aggregated accuracy for each valence level in the iconic memory post-test. Initial availability is represented by α; β represents the amount of information transferred to visual short-term memory for further processing, and τ indicates the duration of information in iconic memory

Decay functions, $\:p\left(t\right)=\:\alpha\:{e}^{\frac{-t}{\tau\:}}+\beta\:$, were fitted to the accuracy as a function of cue delay *t* with the indicator of initial availability in iconic memory α and the amount of information selected for further processing by transferring it to visual working memory β. The parameter τ indicates the duration of availability, meaning a higher value revealing slower decay (Graziano & Sigman, 2008; Lu et al., 2005). An adaptive non-linear least-squares algorithm was used (Dennis et al., 1981). The parameter estimates for each valence level can be seen in Fig. 6. It appears that the initial availability α for positive CSs was lower compared to negative or neutral CSs. The selection parameter β was higher for positive CSs than neutral CSs. The lowest value for the selection parameter β was for negative CSs. The decay parameter τ, on the other hand, was highest for negative CSs compared to neutral and positive CSs, indicating a slower decay for negative CSs in iconic memory. Differences due to valence in the amount of selected information for transferral were statistically significant ($\:{\chi\:}^{2}\left(2\right)=7.99,\:p=\:0.018$, *w* = 0.010) when analysing parameter β based on individually fitted decay functions using a similar algorithm (Levenberg, 1944; Marquardt, 1963). Significant differences could not be confirmed for the parameter α ($\:{\chi\:}^{2}\left(2\right)=5.17,\:p=\:0.075$) nor for parameter τ ($\:{\chi\:}^{2}\left(2\right)=5.31,\:p=\:0.070$).

### Ratings of CSs and iconic memory pre-test

Based on the unexpected main effect of valence in the CS ratings, the ratings were investigated further for ratings of each stimulus rather than their assigned valence. Only the pre-rating was looked at due to being without any prior manipulation. The slightly left-tilted-looking CS of an angle of 157.5° was rated lowest on average (*M* = 9.58, *SD* = 3.43). The highest average rating received the slightly right-tilted-looking stimulus of an angle of 22.5° (*M* = 12.23, *SD* = 3.52). In between were the ratings for the more left-tilted-looking stimulus of an angle of 112.5° (*M* = 10.65, *SD* = 2.97) and for the more right-tilted-looking stimulus of an angle of 67.5° (*M* = 11.23, *SD* = 3.39). A one-way repeated measures analysis of variance for the factor stimulus confirmed a main effect of stimulus, *F*(2.23,87.10) = 5.34; *p* = .005; $\:{\eta\:}_{G}^{2}$ = 0.078. A correction of degrees of freedom was made with ε = 0.744. While there was no significant difference in ratings between the stimuli of 67.5° and 112.5° (*p* =.447), there was a significant difference in ratings for the stimuli of 22.5° and 157.5° with lower ratings for the stimulus of 157.5° (*p* =.007, *dz* = -0.556). The significance level was missed for the ratings of stimuli of 22.5° and 67.5 (*p* = .090), stimuli of 22.5° and 112.5° (*p* = .065), stimuli of 67.5° and 157.5° (*p* = .065), as well as of stimuli of 112.5° and 157.5° (*p* = .065). Due to the possibility that a prior liking might have influenced performance in the iconic memory test, accuracy in the iconic memory pre-test was investigated based on the pre-rating of each stimulus.

One stimulus was selected as the most liked, and one as the least liked stimulus for each participant. The other stimuli were then encoded as the two neutral stimuli. With the encoding of the most (positive equivalent) and least liked (negative equivalent) stimuli, accuracy in the iconic memory pre-test was analysed. Average accuracy was lowest for negatively perceived stimuli in the delay of 493 ms. The highest average accuracy was found for neutrally perceived stimuli in the 17 ms cue-delay condition. The averages and standard deviations can be seen in the table in Appendix B. A repeated-measures analysis of variance for the factors prior perceived valence and delay revealed a main effect of delay, *F*(4,156) = 6.95; *p* < .001; $\:{\eta\:}_{G}^{2}$ = 0.035, but no main effect of prior perceived valence, *F*(2,78) = 0.29; *p* =.728. There was no interaction of prior perceived valence and delay, *F*(6.09,237.46) = 1.24; *p* =.284. Degrees of freedom were corrected with ε = 0.755. Prior perceived valence of stimuli did not affect accuracy in the iconic memory pre-test.

## Discussion

Instead of an interaction of valence and time of rating, only a main effect of valence was observed in CS ratings. A significant interaction would have shown a change in rating, which can be defined as the evaluative conditioning effect (De Houwer, 2007). Still, valence appears to be playing a role in the iconic memory post-test. The shortest response times were observed for positive CSs, and the longest response times were observed for negative CSs. The highest accuracy could be observed for positive CSs in the iconic memory post-test across delays, while accuracy for negative CSs was the lowest. Any possible prior perceived valence of the stimuli used did not show any effect on accuracy in the iconic memory pre-test prior to the evaluative conditioning phase.

The reason for no interaction of valence and time of rating could lie in prior differences in ratings of the stimuli. In particular, the slightly to the left tilted stimulus was perceived on average worse than the slightly to the right tilted stimulus. The orientation of diagonal lines has been observed to influence the liking of products (Schlosser et al., 2016). Product packaging with upward (tilted to the right) diagonal orientations in text or image in an active context was more appealing than packaging with downward (tilted to the left) diagonal orientations or a neutral control. In contrast, this was the other way around when the context was passive or relaxing. While the CS ratings in the current experiment differed slightly in a more positive direction when the lines were tilted upwards, this was not generally significant between upward and downward-oriented CSs. The CS rating task did not provide an active or passive context, which could have heightened a possible effect of orientation on ratings.

Another reason for not observing a significant interaction of valence and time of rating could be due to not having enough power to detect an interaction effect. Figure 3 shows how ratings changed on average between the pre- and post-ratings of CSs. Hence, it is believed that a change in rating has occurred, but this was not detectable on a significant level. Significant differences between ratings for positive and neutral as well as for positive and negative CSs could be observed, which appear to be mainly driven by the CS post-ratings (see Fig. 3). Future experiments should consider increasing the number of participants or changing the design accordingly.

While no significant interaction of valence and time of rating could be observed in CS ratings, valence still appeared to have affected response times and accuracy in the iconic memory post-test. While the shortest response times were observed for positive CSs, response times for negative CSs were the longest. Response times are usually not reported in studies using iconic memory tests. Studies on affective content and visual attention have revealed that attention can be guided by affectiveness and lead to shorter response times for emotional stimuli compared to neutral stimuli (e.g., Öhman et al., 2001; Vuilleumier, 2005). Hence, looking at response times was seen to be important as well. In contrast to shorter visual search times found in the visual study reported by Öhman et al. (2001), in the present iconic memory post-test with conditioned stimuli, the longest response times were observed for negative CSs. It appears that response times in an iconic memory test might be differently affected than search times in visual search tasks. When looking at a study on visual working memory, observations on response times appear a bit similar. Rozovskaya et al. (2016) found quicker response times for positive images when participants had to report if a change had occurred or not. The longest response times were observed for negative images, but the shortest response times were for neutral images (Rozovskaya et al., 2016).

The highest accuracy could be observed for positive CSs, and the accuracy for negative CSs was the lowest. This is in accordance with the findings by Kattner and Green (2019), who also observed the lowest accuracy for negative CSs and the highest for positive CSs. In addition to the author’s findings, differences in accuracy were found for all levels of valence in the current experiment. Similar to Experiment 2a but in contrast to Experiment 2b in the above-mentioned study, no interaction of valence and cue delay was observed. Both the results of the present experiment and the results of Experiment 2a by Kattner and Green (2019) are contrary to the findings by Kuhbandner, Spitzer, and Pekrun (2011) when looking at performance in accordance with valence level. Kuhbandner, Spitzer, and Pekrun (2011) observed the highest accuracy for negative stimuli and the lowest for neutral stimuli. This was the opposite in the present experiment, where the lowest performance was observed for negative CSs and the highest performance for positive CSs. Kattner and Green (2019) observed the lowest accuracy for negative CSs and the highest for positive CSs. Hence, the question arises if conditioned valence has a different level of impact on visual processing or if other factors like visual characteristics of affective stimuli influenced accuracy as well, which then led to the different results in the mentioned iconic memory tests. As both the present study and the experiments reported by Kattner and Green (2019) revealed similar results, it appears to be more likely that more than valence influenced performance in the study Kuhbandner, Spitzer, and Pekrun (2011).

When an affective state is induced in participants instead of stimuli containing the valence aspect, participants were significantly better in an iconic memory test when in a positive state compared to when in a neutral or negative state (Kuhbandner, Lichtenfeld, & Pekrun, 2011). There were no significant differences between the neutral and negative emotional state conditions. Hence, it appears positive inner state as well as positive conditioned valence could have a similar effect on visual processing. Mood has been found to affect working memory, as negative mood can lead to lower memory capacity (Spachtholz et al., 2014) and higher precision in answers (Spachtholz et al., 2014; Xie & Zhang, 2016). One might ask if the higher precision would also apply to iconic memory, but Xie and Zhang (2016) ruled this out. Presentation time can change the likelihood of remembering according to Maljkovic and Martini (2005). Participants were more likely to remember a negative image correctly, when the presentation time was increased. This was not the case for neutral nor positive images, where the probability of remembering an image stayed constant. If this would also apply to iconic memory tests where stimuli are only presented for a very short time, lower accuracy could be explained by less information being accumulated for negative stimuli. This could explain the low parameter β, the indicator for the amount of information transferred to short-term memory, for negative CSs in the iconic memory post-test of the present study.

Interestingly, the shortest response times did not coincide with the lowest accuracy. As mentioned above, the shortest response times and highest accuracy were observed for positive CSs. In contrast, the response times for negative CSs were the slowest, and accuracy was the lowest. Usually, when looking at and discussing response time-accuracy trade-offs, the reasoning is that the shortest response times lead to lower accuracy (see e.g., Schmitt & Scheirer, 1977; Wickelgren, 1977). It is not unusual though to observe shorter response times and higher accuracy at the same time. Galindo et al. (2015) observed the shortest response times in a working memory task for neutral images and also the highest accuracy for neutral images. Response times for positive images were significantly slower and response times for negative images were the slowest on average. Accuracy for both positive and negative images was significantly lower compared to accuracy for neutral images, but performance did not differ between the affective valence conditions (Galindo et al., 2015).

Rensink (2014) states the idea of iconic memory having different layers, which correspond to different layers in visual processing. An item is processed in its basic parts on lower levels, like brightness and orientation, and higher more complex levels like being seen as an individual item and its location in the surrounding area. The detailed lower-level information of the stimulus or its accessibility decays more quickly, while access to higher-level information lasts a bit longer. Rensink (2014) proposes that limitations in accessing certain layers could lead to disruptions in feedback loops of information processing and then lead to more response errors. Rensink (2014) observed different response time limits between different types of tasks, change detection, change report, and static detection. Rensink (2014) reasoned that these differences could be due to the different levels of how much of an individual item has to be processed to respond correctly, which led to the theory of the iconic memory consisting of several layers. In the current study, we see differences in both response time and accuracy in the iconic memory post-test due to conditioned valence. Hence the question arises if the valence conditions were experienced as different tasks, leading to different processing in iconic memory.

A slower decay of information, as it was observed for negative stimuli by Kuhbandner, Spitzer, and Pekrun (2011), could explain why a higher accuracy was observed for negative stimuli in the iconic memory test. Kuhbandner, Spitzer, and Pekrun (2011) also reported a significantly higher amount of information for positive and negative stimuli was transferred to short-term memory. Hence, there was more information available for a longer period for negative stimuli. In the present study, a significantly higher amount of transfer to short-term memory was only observed for positive stimuli, which could explain the higher accuracy given that response times were shorter for positive stimuli and therefore information more likely still available. While decay of information was highest for negative stimuli also in the current experiment, this was not significant. The lower accuracy for negative stimuli could be explained by the lower transfer of information to short-term memory in the first place and the slower response times. Less information was available to respond in trials with negative targets.

Differences in accuracy for the cue-delay conditions were similar to those found in other studies using the iconic memory test (Kuhbandner, Spitzer, & Pekrun, 2011). While differences in accuracy were not observable between all cue delays, the decay of information with prolonged cue delay was still observable, whereby accuracy was highest in the shortest cue-delay condition and lowest in the longest cue-delay condition.

When looking at the decay functions and the parameter estimates, in particular, the picture appears to be close in some parts to the results by Kattner and Green (2019) and to some by Kuhbander, Spitzer et al. (2011). The amount of selected information for transfer to visual working memory, parameter β, was highest for positive CS, which was also found by Kattner and Green (2019). Contrary to the aforementioned study, significant differences between valence levels could be observed in the present study. Kuhbandner, Spitzer, and Pekrun (2011) did not report any differences in the parameter β between positive and negative stimuli but between affective and neutral target conditions.While the initial availability α was found to be either slightly higher for positive stimuli (Experiment 2a in Kattner & Green, 2019) or for negative stimuli (Kuhbandner, Spitzer, & Pekrun, 2011) or for neutral stimuli (current experiment; Experiment 2b in Kattner & Green, 2019), none observed significant differences. Hence, it appears that any differences observed in the accuracy of the iconic memory test are not due to initial availability.

Kattner and Green (2019) found the slowest decay for positive CSs indicated by parameter τ in one experiment (Experiment 2a) and in another experiment, the slowest decay for negative CSs (Experiment 2b). Similarly to the latter experiment and to the observation of parameter τ for negative stimuli by Kuhbandner, Spitzer, and Pekrun (2011), the decay parameter τ for negative CSs was slightly but not significantly higher compared to the decay parameter for neutral and positive CSs. Not observing significant differences due to valence for the decay parameter τ could be due to a lack of power. Future studies intending to investigate if conditioned valence can impact decay of information in iconic memory should take care of having enough statistical power in their design to be able to observe possible differences due to conditioned valence. Throughout the current study, differences due to valence were below expectations when looking at positive versus neutral and negative versus neutral valence conditions compared to the expected effect size of *dz* = 0.537 (Hofmann et al., 2010).

## Conclusion

Overall, it appears that it could be shown again that conditioned valence can be used as a method to investigate valence as a potential factor in visual processing. It also appears that conditioned valence can affect visual processing, and this can be replicated using different stimuli and different conditioning methods as well as different stimuli presentations in the iconic memory test. In addition, it seems to be more certain now, that initial availability in iconic memory is not the key factor for higher accuracy in the iconic memory test. The amount of information transferred to short-term memory appears to be a likely candidate for explaining differences due to valence. Prolonged decay of information could be another factor when looking at prior studies, but this could not be confirmed in the present study. Further research should continue investigating the stability of differences in decay depending on valence levels.

## Data Availability

Data is available on osf.io: https://osf.io/jgrh5/?view_only=4b884cf24518437a9b924586fb9241b4.

## Appendix A

**Table:**

Conditioned valence	Cue delay (ms)	M	SD	SEM
Negative	17	1565.77	395.08	67.76
Neutral	17	1331.69	359.78	56.89
Positive	17	1284.30	321.58	50.84
Negative	68	1517.32	422.80	66.85
Neutral	68	1339.54	355.59	56.94
Positive	68	1199.75	327.77	52.48
Negative	221	1537.25	433.52	75.47
Neutral	221	1267.72	428.19	67.70
Positive	221	1215.62	360.53	57.73
Negative	493	1391.49	576.02	97.36
Neutral	493	1338.60	426.34	67.41
Positive	493	1245.34	359.58	57.58
Negative	1003	1551.56	433.62	75.48
Neutral	1003	1314.97	372.63	58.92
Positive	1003	1197.80	437.52	70.98

*Note*: Average response time (ms) for each valence level and cue-delay in the iconic memory post-test. The standard deviation and standard error of the mean are reported as well

## Appendix B

**Table:**

Perceived valence	Cue delay (ms)	M	SD	SEM
Negative	17	0.38	0.23	0.04
Neutral	17	0.42	0.20	0.03
Positive	17	0.41	0.23	0.04
Negative	68	0.36	0.22	0.03
Neutral	68	0.35	0.20	0.03
Positive	68	0.40	0.25	0.04
Negative	221	0.33	0.19	0.03
Neutral	221	0.37	0.15	0.02
Positive	221	0.32	0.23	0.04
Negative	493	0.24	0.15	0.02
Neutral	493	0.31	0.13	0.02
Positive	493	0.32	0.22	0.03
Negative	1003	0.36	0.20	0.03
Neutral	1003	0.32	0.11	0.02
Positive	1003	0.33	0.21	0.03

*Note*: Average proportion correct for encoded perceived valence of stimuli in each cue-delay condition in the iconic memory pre-test. The standard deviation and standard error of the mean are reported as well
